# Analysis of Variants' Dynamic Using the CLIMB Database in COVID‐19 Patients Admitted to Hospitals of Barts Health NHS Trust

**DOI:** 10.1002/jmv.70402

**Published:** 2025-05-24

**Authors:** Concetta Piazzese, Sophie Williams, Adam Brentall, Beatrix Kele, Jon Bible, Kathryn Harris, Teresa Cutino‐Moguel

**Affiliations:** ^1^ PHURI Queen Mary University of London London England UK; ^2^ DERI Queen Mary University of London London England UK; ^3^ Barts Life Sciences Barts Health NHS Trust London England UK; ^4^ Wolfson Institute of Population Health Queen Mary University of London London England UK; ^5^ Respiratory Virus Unit UK Health Security Agency London England UK; ^6^ Department of Virology, Division of Infection Barts Health NHS Trust London England UK

**Keywords:** CLIMB‐COVID database, lineage alpha, SARS‐CoV‐2

## Abstract

The COVID‐19 pandemic, caused by SARS‐CoV‐2, has led to significant global health challenges. This study analyzes the dynamics of SARS‐CoV‐2 variants among patients admitted to Barts Health National Health Service (NHS) Trust hospitals using data from the CLIMB‐COVID decentralized digital infrastructure allowing precise identification of SARS‐CoV‐2 variants. A total of 423 patients admitted between October 2020 and March 2021 were included in the study and divided into two groups: the alpha lineage group, which comprised the B.1.1.7 variant, and the other lineages group, which included all other variants. Whole‐genome sequencing of SARS‐CoV‐2 genomes was conducted using the COVID‐CLIMB pipelines. Clinical outcomes, such as mortality rates and deterioration within 28 days, were analyzed. To ensure robust findings, analyzes were adjusted for confounding factors, including age and comorbidities. Our findings revealed a significant increase in mortality with age for the alpha lineage and other lineages. The study underscores the importance of age adjustment in clinical studies to accurately assess the impact of different variants. Consistent genomic sequencing and data completeness are crucial for obtaining reliable results and guiding public health responses. These insights are vital for improving patient outcomes and providing a truthful picture of the pandemic, informing both current and future healthcare strategies.

## 1 Introduction

In 2019, a novel disease, named COVID‐19, emerged in China and weeks later a new beta‐coronavirus (SARS coronavirus 2, or SARS‐CoV‐2) was identified as the causal agent [1]. In the United Kingdom, the first cases of humans infected with SARS‐CoV‐2 were reported in January 2020 [2]. The National Health Service (NHS) and similar healthcare systems were ill‐prepared in monitoring the spread of infection with SARS‐CoV‐2 in their populations. At immense societal costs, test and trace systems were developed and employed at scale to provide accurate data on the prevalence of infection across the UK population. In short, population surveillance for infection by SARS‐CoV‐2 has been based on the following pillars:

- Reporting of cases by the UK Health Security Agency (UK‐HSA).
- Sentinel screening in a random population sample by the Office for National Statistics (ONS), followed by molecular characterization.
- Outbreak investigation via NHS Test and Trace.

All the above systems are based on verification of the infectious agent by polymerase chain reaction (PCR) based detection of viral nucleic acids or by a lateral flow test which detects viral proteins.

Deciphering the genetic material found in an organism or virus has played an important role in understanding of the outbreak characteristics and transmission pattern during the SARS‐CoV‐2 pandemic [3]. Local outbreak investigation is now possible using sequencing technologies [4] that can give valuable information on the epidemiology of the virus [5, 6]. A national consortium, named the COVID‐19 Genomics UK (COG‐UK) was established in March 2020 [7] to develop a decentralized digital infrastructure, called the Cloud Infrastructure for Big Data Microbial Bioinformatics database (CLIMB). This infrastructure could provide whole‐genome virus sequencing and sample‐associated metadata analysis for local NHS centers and the UK government. As of March 2022, more than 2 million genome sequences have been processed by the CLIMB‐COVID centralized data environment [8]. The sequences and the limited metadata available in the infrastructure have been widely used by researchers to perform analyzes on SARS‐CoV‐2 virus genome mutations and variants circulating in the United Kingdom [9, 10, 11, 12, 13, 14].

In March 2020, the Barts Health COVID Research Delivery Group began to assemble a comprehensive data set including entire time course data of patients hospitalized in five Barts Health Hospital sites that serve a community of 2.5 million people with rich diversity drawn from 97 nations. Every day routinely collected health data from Oracle Millennium were assembled and curated using natural language processing on patients hospitalized with a diagnosis of COVID‐19 [15].

As part of a national‐scale genomic surveillance strategy, COVID sample‐associated metadata collected from Barts Health patients were also sequenced and analyzed using a decentralized digital infrastructure (CLIMB‐COVID) supporting outbreak/pandemic response at local, regional, national, and global levels.

The aim of this study is to evaluate the genomic analysis of positive samples collected from October 2020 to March 2021 in the east London area. The analysis was performed by assessing the contribution of whole‐genome sequencing of SARS‐CoV‐2 genomes performed through COVID‐CLIMB pipelines (https://github.com/COG-UK) combined with the health data of COVID patients hospitalized in five Barts Health Hospitals extracted from Oracle Millennium.

## 2 Material and Methods

### 2.1 Study Population and Data Collection

A total of 5467 adult patients (> 17 years old) admitted through the emergency department in three Barts Health Hospital sites (Newham University Hospital, Royal London Hospital, Whipps Cross University Hospital) between 01 October 2020 to 01 March 2021 and with a confirmed PCR SARS‐CoV‐2 test result within a 7 d window of admission were initially identified.

Among these, raw genomic sequencing was performed for 1068 patients, which were uploaded to the CLIMB‐COVID database and analyzed using the machine learning‐based assignment algorithm PangoLEARN (https://github.com/cov-lineages/pangolin, database version 2021‐11‐04) which assigns cases to a lineage using Pango nomenclature [16]. Given that lineage designation of sequences with low genome coverage is not possible, samples of patients with genome completeness less than 93% were not processed by the pipelines available in the CLIMB server and were excluded from this study. This resulted in 533 patients meeting the quality threshold for further processing.

Data of 533 patients processed by the pipelines of CLIMB infrastructure was collated with available metadata (demographic information, comorbidities, vital signs and clinical outcomes) collected from clinical records [17]. Patients with missing data were removed from the study, resulting in a final cohort of 423 patients. We categorized the cohort into two groups: the lineage alpha group, comprising patients infected with the alpha variant (B.1.1.7), and other lineages group, comprising patients infected with other variants such as B.1 or B.1.* or B.1.1.* (and excluding B.1.1.7) or AD.2 or C.16 or P.2 or W.3, where XX.* indicates all sequences starting with the characters ‘XX.’

Data on mortality rates, and deterioration within 28 days were specifically analyzed for the two groups.

### 2.2 Statistical Analysis

Descriptive statistics were used to summarize patient characteristics. The prognosis of COVID‐19 patients was assessed by examining the mortality rates (based on the ISARIC 4 C Mortality (4C–M) score [18] and deterioration rates (the ISARIC 4C–D score [19] across different age groups and lineage groups. Kaplan‐Meier survival curves were plotted to visually compare the mortality and deterioration rates over the 28‐day period for the different age groups.

We employed proportional hazard regression models to evaluate the differences in 28‐day mortality and deterioration rates among COVID‐19 patients across different viral lineages and age groups. Missing data were addressed using multiple imputation by chained equations (MICE), with estimates combined according to Rubin's rules to provide 95% confidence intervals (CIs). In particular, the following models were applied for mortality:

- Model 1: Adjusted for the ISARIC 4C–M, examining the impact of viral lineage (lineage alpha vs. other lineages) on mortality rates. The interaction between the 4C–M score and lineage was also assessed.
- Model 2: Adjusted for age groups (according to the ISARIC 4C–M score categories) and the National Early Warning Score (NEWS) 2 score [20, 21], further exploring the mortality differences between lineage alpha and. other lineages.

Deterioration models incorporate time‐dependent effects to account for varying risks during different phases post‐admission. Specifically, the models distinguish between two time periods: the initial 12 h following admission (entry), and the period beyond 12 h post‐admission (subsequent). This distinction is crucial as many patients are admitted directly to the ICU at the time of admission, resulting in different risk profiles for deterioration during these time frames. In particular, the following models were applied for deterioration:

- Model 3: Focused on the 28‐day deterioration rates based on ISARIC 4C–D score, considering initial and subsequent events like ICU admission or death, adjusting for age and NEWS2 score.
- Model 4: Examined the interaction effects between age, NEWS2 score, and viral lineage on deterioration rates.

Models 1 and 3 were extended to consider difference by ethnicity, by including an additional term (and interaction by lineage) for ethnicity.

Continuous and binary variables in patients categorized into two groups (alpha lineage and other lineages) were analyzed with *t*‐tests to compare the means between the two groups. Additionally, adjustments for age were made using linear regression models to account for potential confounding effects of age on the observed difference.

## 3 Results

### 3.1 Patient Demographics and Variants Distribution

The study included a total of 423 patients, with 311 in the alpha lineage group and 112 in the other lineages group. Baseline characteristics and deprivation for lineage alpha and the other lineages are summarized in Table 1.

**Table 1:** Demographic breakdown of two different lineages across various categories such as ethnicity, age, sex, and deprivation quintile.

	Lineage alpha (*N*, %)	Other lineages (*N*, %)
Ethnicity		
White	93/311 (30%)	33/112 (29%)
Asian (Indian, Bangl, Pakst)	112/311 (36%)	57/112 (51%)
Black, mixed, or other	77/311 (25%)	18/112 (16%)
Unknown	29/311 (9%)	4/112 (4%)
Age		
18–49 y	61/311 (20%)	23/112 (21%)
50–59 y	56/311 (18%)	14/112 (12%)
60–69 y	73/311 (23%)	23/112 (21%)
70–79 y	52/311 (17%)	24/112 (21%)
80 y+	69/311 (22%)	28/112 (25%)
Unknown	0/311 (0%)	0/112 (0%)
Sex		
Male	167/311 (54%)	64/112 (57%)
Female	144/311 (46%)	48/112 (43%)
Unknown	0/311 (0%)	0/112 (0%)
Deprivation (quintile)		
1	114/311 (37%)	43/112 (38%)
2	137/311 (44%)	52/112 (46%)
3	30/311 (9%)	11/112 (10%)
4	21/311 (7%)	4/112 (4%)
5	7/311 (2%)	2/112 (2%)
Unknown	2/311 (1%)	0/112 (0%)

*Note:* The numbers indicate the count (*N*) and the percentage (%) of individuals within each category for both lineage alpha and other lineages.

**Figure 1:**
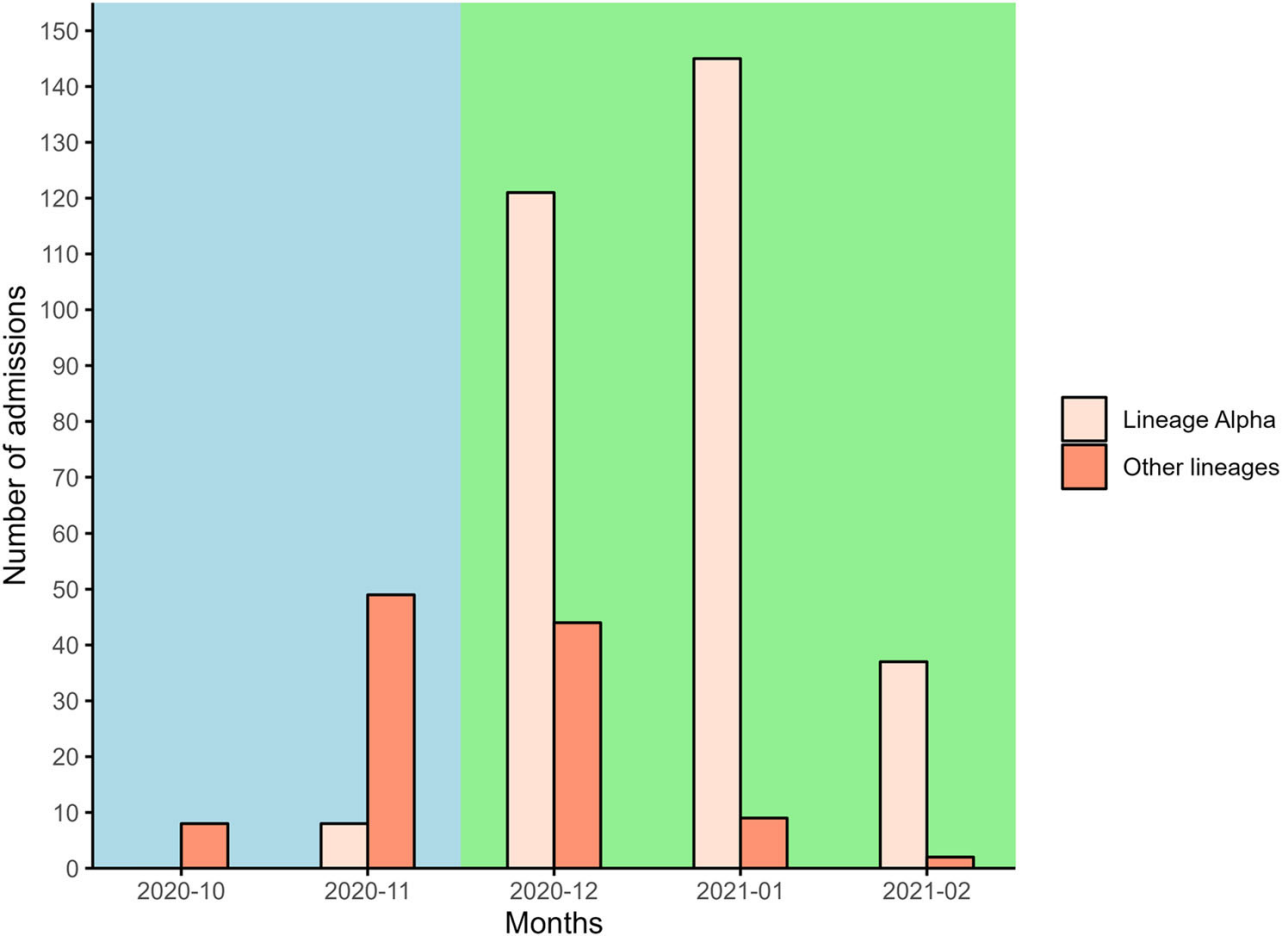
Number of admission of patients per month for lineage alpha and other lineages across two waves of an outbreak. Lineage alpha (beige bars) comprises patients infected with the alpha variant (lineage B.1.1.7), while other lineages (coral bars) include patients infected with other lineages such as B.1, B.1.*, B.1.1.* (excluding B.1.1.7), AD.2, C.16, P.2, or W.3, where XX.* indicates all sequences starting with the characters “XX”. The cyan background highlights the months (October 2020–November 2020) included in the first wave and the green background indicates the months (December 2020–February 2021) included in the second wave. The data shows an increase in admissions coinciding with the epidemiological shift toward the alpha variant, reflecting its higher transmission rates and significant impact on the pandemic's progression.

The occurrences of various COVID‐19 lineages grouped by broader lineage categories and their distribution across different waves is shown in Table 2 and Figure 1. The data highlights the predominance of the alpha lineage compared to other lineages. The alpha lineage (B.1.1.7) was the most prevalent, accounting for 311 cases (74% of the total). The B.1.1.* lineage group, encompassing several sub‐lineages, was the second most common with 112 cases (26% of the total). Other variants such as B.1, AD.2, C.16, and W.3 were much less frequent.

**Table 2:** Occurrence counts of various COVID‐19 lineages grouped by broader lineage categories.

Variant	Occurrences	Lineage group
B.1.1.7	311	Alpha
B.1.1.*	102	Other
B.1	4	Other
AD.2	3	Other
C.16	1	Other
W.3	2	Other

### 3.2 Clinical Outcomes

Table 3 presents the observed and expected 28‐day mortality rates for different age groups and viral lineages. The expected mortality rates (E) were based on the mean 4C–M score (with 95% CI), while the observed mortality rates (O) were derived from clinical data. For both lineages groups, the expected and the observed 28‐day mortality rates, increased with age, with the highest rates in the 80+ age group. For lineage alpha, the observed mortality was higher than the expected mortality only in the 18 to 49 and 60 to 69 age groups, while for other lineages, the observed mortality was higher than expected only in the 60 to 69 and the 70 to 79 age groups. Furthermore, the mortality rates increase with age, with other lineages exhibiting slightly higher rates only in the 60 to 69 and the 70 to 79 age groups.

**Table 3:** Observed and expected mortality within 28 days, by age group and lineages.

	18–49 y	50–59 y	60–69 y	70–79 y	80 y+
E 28 d mortality* (Lineage alpha)	4% (4%–5%)	12% (10%–14%)	23% (20%–26%)	38% (33%–43%)	44% (39%–48%)
E 28 d mortality* (Other lineages)	5% (4%–7%)	10% (7%–14%)	18% (14%–23%)	38% (31%–46%)	41% (34%–48%)
O 28 d mortality* (Lineage alpha)	8% (1%–15%)	9% (1%–16%)	29% (17%–39%)	31% (17%–43%)	42% (29%–53%)
O 28 d mortality* (Other lineages)	0% (0%–0%)	7% (0%–20%)	41% (16%–58%)	42% (18%–58%)	39% (18%–55%)
Died (n) (Lineage alpha)	5	5	20	16	28
Died (n) (Other lineages)	0	1	9	10	11
At risk [days] (Lineage alpha)	1567	1470	1566	1147	1323
At risk [days] (Other lineages)	644	377	518	503	583
Mortality rate (Lineage alpha)	3.2 (1.0–7.4)	3.4 (1.1–7.9)	12.8 (7.8–19.7)	13.9 (8.0–22.6)	21.2 (14.1–30.6)
Mortality rate (Other lineages)	0.0 (0.0–5.7)	2.7 (0.1–14.8)	17.4 (7.9–33.0)	19.9 (9.5–36.5)	18.9 (9.4–33.7)

*Note:* E is the expected 28 days mortality based on the mean 4C–M score (95% CI); O is observed at 28 days from Kaplan‐Meier estimations; rate (95% CI) is per 1000 person/days.

The Kaplan‐Meier survival curves representing the 28‐day mortality rates (Supporting Information Figure F1) and 28‐day deterioration rates (Supporting Information Figure F2) for patients across different age groups shows that there is no statistically significant difference in the 28‐day mortality and deterioration rates between patients infected with lineage alpha or other lineages.

### 3.3 Proportional Hazard Regression Models

Model 1 assessed the impact of viral lineage on mortality rates by adjusting for the ISARIC 4C–M score, which accounts for clinical and demographic factors influencing mortality. The results indicated that the relative hazard was 0.73 (95% CI: 0.54–0.92) for lineage alpha and 1.19 (95% CI: 0.78–1.83) for other lineages. While this suggests a higher mortality for other lineages than for lineage alpha, it also indicates that the increase in mortality risk for other lineages is not statistically significant. The interaction term between the 4C–M score and other lineages was not statistically significant, suggesting that the 4C–M score had an effect on mortality risk in a similar way across both groups. The observed differences in mortality are most likely to be influenced by a combination of lineage‐specific effects and other patient‐level factors captured by the 4C–M score, rather than by lineage effects alone.

In model 2, the relative hazard for different age groups and NEWS2 scores, adjusted for other lineages, is detailed in Table 4. The results show that younger age groups (< 50 years and 50–59 years) had lower estimates (0.12 and 0.09, respectively), compared to the reference group (80+ years), suggesting a reduced likelihood of the outcome compared to older age groups. The NEWS2 score showed a significant positive association with the outcome, with an estimate of 1.18 per unit increase (95% CI: 1.10–1.27). The relative hazard for other lineages was 1.24 (95% CI: 0.80–1.90), which was not significantly different from lineage alpha.

**Table 4:** Hazard ratios (HR) with 95% confidence intervals (CI) for mortality, adjusted for age groups, NEWS2 score, and viral lineage, compared to the reference group (80+ Years).

Parameter	Estimate (95% CI)
70–79 y	0.65 (0.39–1.08)
60–69 y	0.54 (0.33–0.89)
50–59 y	0.12 (0.05–0.29)
< 50 y	0.09 (0.03–0.22)
NEWS2 (per unit)	1.18 (1.10–1.27)
Other lineages	1.24 (0.80–1.90)

In model 3, the adjusted difference between lineage alpha and other lineages for deterioration was examined using the ISARIC 4C–D score (Table 5). The estimates show that for lineage alpha, 4C–D levels decrease from entry (0.71) to subsequent stages (0.44). In contrast, for other lineages, 4C–D levels increase from entry (0.82) to subsequent stages (1.61). The interaction effects were not statistically significant, with confidence intervals overlapping zero, suggesting no meaningful interaction between the variables.

**Table 5:** Adjusted difference between lineage alpha and other lineages for deterioration using the ISARIC 4C–D score (model 3).

Parameter	Estimate (95% CI)
4C–D, entry (Lineage alpha)	0.71 (0.45–0.97)
4C–D, subsq (Lineage alpha)	0.44 (0.12–0.77)
4C–D, entry (Other lineages)	0.82 (0.49–1.37)
4C–D, subsq (Other lineages)	1.61 (0.86–3.01)
4C–D, entry (Interaction)	0.20 (−0.31–0.71)
4C–D, subsq (Interaction)	0.12 (−0.53–0.76)

*Note:* The model distinguishes between two time periods: the initial 12 h following admission (entry) and the period beyond 12 h post‐admission (subsq).

Table 6 shows the age‐adjusted differences between lineage alpha and other lineages for deterioration (model 4). For individuals aged 50–59 and 60–69 years with lineage alpha, the HR for the initial 12 h is 2.58 and 2.62, respectively, suggesting that the risk of clinical deterioration during this period is more than twice that of the reference group (18–49 years with Lineage Alpha). However, beyond 12 h post‐admission, the HR drops, indicating a non‐significant reduction in risk. The HR increases beyond 12 h post‐admission in older age groups (70–79 years and 80+ years). For other lineages, the effects are generally less pronounced, with wide confidence intervals and no significant estimates across any age group.

**Table 6:** Age‐adjusted difference between lineage alpha and other lineages for deterioration (model 4) with the reference group (18–49 y lineage alpha).

Parameter	Estimate (95% CI)
50–59 y, entry (Lineage alpha)	2.58 (1.28–5.20)
50–59 y, subsq (Lineage alpha)	0.84 (0.28–2.53)
60–69, entry (Lineage alpha)	2.62 (1.33–5.15)
60–69, subsq (Lineage alpha)	1.59 (0.65–3.87)
70–79 y, entry (Lineage alpha)	1.14 (0.50–2.59)
70–79 y, subsq (Lineage alpha)	1.65 (0.69–3.95)
80 y+, entry (Lineage alpha)	0.30 (0.08–1.06)
80 y+, subsq (Lineage alpha)	1.01 (0.40–2.56)
18–49 y, entry (Other lineages)	0.00 (0.00–0.00)
18–49 y, subsq (Other lineages)	0.00 (0.00–0.00)
50–59 y, entry (Other lineages)	0.63 (0.16–2.54)
50–59 y, subsq (Other lineages)	1.30 (0.17–9.94)
60–69, entry (Other lineages)	0.19 (0.04–0.90)
60–69, subsq (Other lineages)	1.20 (0.26–5.53)
70–79 y, entry (Other lineages)	0.73 (0.16–3.32)
70–79 y, subsq (Other lineages)	1.24 (0.26–5.82)
80 y+, entry (Other lineages)	0.51 (0.04–6.12)
80 y+, subsq (Other lineages)	0.81 (0.15–4.35)

*Note:* The model distinguishes between two time periods: the initial 12 h following admission (entry) and the period beyond 12 h post‐admission (subsq). For individuals aged 80 years and older with other lineages, the HR for the initial 12 h is 0.00 (95% CI: 0.00 to Inf), indicating no recorded events during this period.

Table 7 presents the adjusted mortality risk by ethnic group, considering the ISARIC 4C–M score and overall group effect. The adjusted odds ratios (ORs) for mortality in various ethnic groups were compared to the reference group (White ethnicity with lineage alpha). The estimates with other lineages for the White, Asian and Unknown ethnic groups and other lineages were higher compared to the same ethnicities with lineage alpha. The Unknown ethnic group with non‐alpha lineages, showed the highest estimate for 4C–M at 5.59 (95% CI: 0.80–39.27).

**Table 7:** Adjusted estimates and confidence intervals for mortality across different ethnic groups, comparing other lineages with the reference group (white ethnicity, lineage alpha).

Parameter	Estimate (95% CI)
4C–M, Ethnicity A–White (Other lineages)	1.22 (0.61–2.44)
4C–M, Ethnicity B–Asian (Lineage alpha)	1.09 (0.66–1.80)
4C_M, Ethnicity B–Asian (Other lineages)	1.23 (0.48–3.16)
4C–M, Ethnicity C–Black, mixed, or other (Lineage alpha)	0.95 (0.53–1.70)
4C–M, Ethnicity C–Black, mixed, or other (Other lineages)	1.19 (0.11–12.57)
4C–M, Ethnicity D–Unknown (Lineage alpha)	0.43 (0.13–1.45)
4C–M, Ethnicity D–Unknown (Other lineages)	5.59 (0.80–39.27)

*Note:* The calibration slope of the ISARIC 4C–M prognostic score indicates the model's predictive accuracy.

The ISARIC 4C–D prognostic score (Supporting Information Table S1) shows more variable results with the estimates increasing in the period beyond 12 h post‐admission only for the Asian and Black, Mixed, or Other ethnicities with other lineages. Individuals with Unknown ethnicity showed a high risk at entry for lineage alpha, with a wide confidence interval for other lineages beyond 12 h post‐admission.

### 3.4 Prognostic Factor Summaries

Supporting Information Table S2 shows summary statistics for various continuous variables in patients grouped by lineage (lineage alpha and other lineages). Urea, creatinine, systolic blood pressure, and pulse were not statistically significant before age adjustment when comparing lineage alpha and other lineages but became significant after adjusting for age. Conversely, respiratory rate, oxygen saturation on air, and NEWS2 [22] were statistically significant before age adjustment but lost significance after age adjustment. General oxygen saturation, albumin, and National Cancer Institute (NCI) Comorbidity Index [23] were statistically significant between lineages before and after adjusting for age.

Summary statistics for selected binary variables categorized by lineage groups (lineage alpha and other lineages) are shown in Supporting Information Table S3. Among these, Metabolic Disease showed a significant difference between lineage alpha and other lineages after adjusting for age and Diabetes Mellitus with complications was statistically significant before and after adjusting for age.

## 4 Discussion

Our study assessed the clinical outcomes and demographic characteristics of patients infected with different COVID‐19 variants, primarily focusing on the alpha lineage (B.1.1.7) and comparing it to other lineages. Our findings indicate higher, though not statistically significant, rates of mortality and deterioration for patients infected with the lineage alpha which was first detected in our population in November 2020 and was the dominant strain by December 2020. These higher rates are consistent with previous studies that have highlighted the increased transmissibility and potential severity of the Alpha lineage [24, 25, 26, 27].

The main strength of this study is the linkage between granular clinical information collected in hospitals and the detailed genomic sequencing obtained from the COVID‐CLIMB infrastructure, a decentralized digital environment to provide whole‐genome virus sequencing and sample‐associated metadata analysis. The reproducible pipelines integrated within CLIMB‐COVID ensured were accurately and consistently identified using Pango nomenclature. Using the CLIMB‐COVID infrastructure allowed us to generate robust and reliable results and a clear picture of how variants evolved and spread across the country. This makes our work stand out from studies that didn't link these data sources. The methodologies used here not only emphasize the importance of systematic and reproducible approaches but also offer a practical and adaptable framework for studying the clinical impact of future variants. Additionally, the inclusion of diverse demographic groups enhanced the generalizability of our findings. This study adds to the tools available for pandemic preparedness in the United Kingdom and elsewhere.

The demographic analysis in the population analyzed showed that the alpha lineage had a slightly younger median age than for other lineages (64 years old compared to 66 years old). There was a higher proportion of male patients in both groups, but more so for other lineages (62% compared to 55% in the alpha group). Ethnically, the distribution was somewhat similar, with the majority being White or Asian. The alpha lineage had a higher percentage of patients from more deprived quintiles compared to the other lineages, suggesting a possible socioeconomic factor in the spread of this variant.

The predominance of the alpha variant in the second wave (303 out of 311 cases) compared to the first wave (8 out of 311 cases) underscores the rapid transmissibility and possible fitness advantage of this variant [28, 29]. This marked difference between the two waves, however, cannot be attributed solely to the circulating lineages. Other critical factors, such as the availability of vaccines and the introduction of new therapeutics, also played significant roles in shaping the epidemiological landscape during these periods. The second wave occurred at a time when vaccines were beginning to be rolled out on December 8, 2020 to frontline healthcare staff and the highest risk individuals [30], potentially altering the dynamics of variant spread and infection severity. Given that our study period spans from October 2020 to the end of March 2021, it is highly unlikely that any of the patients included in our study were vaccinated during this time. This is further supported by a study by Bernal et al. [31], analyzing data from 8 December 2020 to 19 February 2021 in England, found that a single vaccine dose substantially reduced the risk of severe COVID‐19 outcomes, including hospitalization and death. Although we lack direct data on vaccination status, given the limited early vaccine rollout and the fact that widespread vaccination coverage was only achieved later, any potential confounding effect from vaccination on our results is expected to be minimal. Improvements in clinical management and the availability of therapeutics may have influenced the observed patterns. Thus, while the dominance of the alpha variant highlights its transmissibility and fitness advantage, it is essential to consider these other factors in conjunction with the lineage data to fully understand the differences between the two waves. The varied and less consistent patterns of occurrence of other variants, such as B.1.1.*, B.1, AD.2, P.2, C.16, and W.3, likely reflect the complex interplay of these factors, influencing not only the spread of specific lineages but also the overall impact of the pandemic during different periods.

The analysis of expected and observed 28‐day mortality rates across different age groups for lineage alpha and other lineages reveals significant increase in mortality with age. Both lineages show higher observed mortality rates than expected, especially among older age groups. Despite this trend, there are no significant differences in mortality rates between lineage alpha and other lineages within the same age groups.

Proportional hazard regression models highlighted several critical points. Model 1 assessed the impact of viral lineage on mortality rates, adjusted for the ISARIC 4C–M score. The results suggested a higher mortality for other lineages compared to lineage alpha, though this increase was not statistically significant, and no differential impact of the 4C–M score across lineages was observed.

In model 2, the relative hazard for different age groups and NEWS2 scores, adjusted for other lineages indicated that the relative hazard for mortality in other lineages was not significantly different from that for lineage alpha, suggesting no substantial difference in mortality risk between the two viral lineages.

The results from model 3, as detailed in Table 5, provide insights into how the ISARIC 4C–D score differentially impacts the risk of deterioration between COVID‐19 viral lineages. The findings underscore the importance of early assessment and monitoring using tools like the 4C–D score where higher scores indicate a greater risk of deterioration.

Results from model 4 underscore the complex interplay between age, viral lineage, and timing in influencing the risk of clinical deterioration among patients. The findings confirms that early interventions may be crucial for younger age groups with lineage alpha to mitigate risks associated with disease progression [29, 32]. Moreover, while other lineages generally show comparable risk levels to lineage alpha across different age groups, further investigation is needed to understand the specific factors contributing to the observed differences in clinical outcomes.

In our population, the predictive performance of the ISARIC 4C–M score shows that, when compared to the White ethnic group, the other ethnic groups do not exhibit statistically significant differences in mortality risk across both lineages, except for variability observed within the Unknown ethnic group.

Ethnicity‐specific analyzes with ISARIC 4C–D score showed more variable results, with the estimates increasing beyond 12 h post‐admission specifically for patients of Asian and Black, Mixed, or Other ethnicities with other lineages.

When analyzing the interplay between various clinical and demographic factors across two lineage groups, it is noteworthy that certain variables became significant after adjusting for age while other variables lost significance after adjustment. This indicates that age plays a crucial role in the baseline differences between the two lineage groups for these parameters. It suggests that older patients in one lineage might have inherently different clinical profiles compared to the other, thereby highlighting and confirming the importance of adjusting for age in clinical studies [33] to obtain more accurate results.

For binary variables, diabetes mellitus with complications showed a significant difference between the two lineages, even after age adjustment. This persistence of significance implies a robust difference in the prevalence of diabetes mellitus with complications between the two groups, independent of age. It points to a potential genetic or environmental factor specific to one lineage that increases the risk of Diabetes Mellitus with complications. Other binary variables, such as metabolic disease, did show significant differences between lineage alpha and other lineages after adjusting for age. Overall, these findings emphasize the need for tailored clinical management and further research into the genetic and environmental factors contributing to these differences.

The study's findings should be interpreted in the context of several limitations inherent in the data. Firstly, the sample size, while substantial, may not fully represent the broader population, potentially limiting the generalizability of the results. Additionally, more than 50% of patients were removed from the analysis because they did not pass the quality check in the CLIMB database.

Data completeness was another significant issue, as the presence of missing values for certain clinical variables might have affected the robustness of the statistical analyzes. Although efforts were made to adjust for age and other confounding factors, residual confounding may still exist due to unmeasured variables, such as socioeconomic status or access to healthcare, which were not accounted for in the models. More rigorous and consistent genome sequencing protocols across all sample collection sites would help in achieving higher genome coverage and completeness. Additionally, implementing standardized data collection practices for clinical variables and patient demographics would reduce the incidence of missing or inconsistent data.

Another limitation of our study is the lack of direct data on the vaccination status of patients. However, given the timeline of vaccine rollout and the timeframe considered in this study, it is unlikely that vaccination had a significant impact on our results.

## 5 Conclusion

Our study highlights the significant clinical and demographic differences between patients infected with the alpha lineage and those with other COVID‐19 lineages. The findings underscore the importance of adjusting for age in clinical studies and the need for tailored clinical management strategies. Enhanced data completeness and consistent genomic sequencing are crucial for future research to provide more robust and comprehensive insights into the impact of different SARS‐CoV‐2 variants.

## Author Contributions

**Concetta Piazzese:** contributed to data collection, data curation, methodology, formal analysis, investigation, interpretation of data, and was responsible for writing the original draft as well as reviewing and editing the manuscript. **Sophie Williams:** was involved in conceptualization, supervision, interpretation of data, and writing – review and editing. **Adam Brentall:** contributed to formal analysis, while. **Beatrix Kele:** was responsible for data collection and data curation. **Jon Bible** and **Kathryn Harris:** participated in data collection and writing – review and editing. **Teresa Cutino‐Moguel:** was involved in conceptualization, supervision, interpretation of data, and writing – review and editing. All authors read and approved the final article.

## Conflicts of Interest

The authors declare no conflicts of interest.

## Supporting information

Supplementary material.

## Data Availability

Relevant data supporting the key findings of this study are available within the article and its Supplementary Information files. All other data sets and source code for statistical analyzes generated during this study are available from the corresponding authors upon reasonable request.

## References

[1] J. W. Tang , P. A. Tambyah , and D. S. C. Hui , “Emergence of a Novel Coronavirus Causing Respiratory Illness From Wuhan, China,” Journal of Infection 80, no. 3 (2020): 350–371. 10.1016/j.jinf.2020.01.014 PMC7127306 32001309

[2] P. J. Lillie , A. Samson , A. Li , et al., “Novel Coronavirus Disease (Covid‐19): The First Two Patients in the UK With Person to Person Transmission,” Journal of Infection 80, no. 5 (2020): 578–606. 10.1016/j.jinf.2020.02.020 PMC7127394 32119884

[3] B. B. Oude Munnink , N. Worp , D. F. Nieuwenhuijse , et al., “The Next Phase of SARS‐CoV‐2 Surveillance: Real‐Time Molecular Epidemiology,” Nature Medicine 27, no. 9 (2021): 1518–1524. 10.1038/s41591-021-01472-w 34504335

[4] L. W. Meredith , W. L. Hamilton , B. Warne , et al., “Rapid Implementation of SARS‐CoV‐2 Sequencing to Investigate Cases of Health‐Care Associated COVID‐19: A Prospective Genomic Surveillance Study,” Lancet Infectious Diseases 20, no. 11 (2020): 1263–1271. 32679081 10.1016/S1473-3099(20)30562-4 PMC7806511

[5] M. Lucey , G. Macori , N. Mullane , et al., “Whole‐Genome Sequencing to Track Severe Acute Respiratory Syndrome Coronavirus 2 (SARS‐CoV‐2) Transmission in Nosocomial Outbreaks,” Clinical Infectious Diseases 72, no. 11 (2021): e727–e735. 32954414 10.1093/cid/ciaa1433 PMC7543366

[6] L. B. Snell , C. L. Fisher , U. Taj , et al., “Combined Epidemiological and Genomic Analysis of Nosocomial SARS‐CoV‐2 Infection Early in the Pandemic and the Role of Unidentified Cases in Transmission,” Clinical Microbiology and Infection 28, no. 1 (2022): 93–100. 34400345 10.1016/j.cmi.2021.07.040 PMC8361005

[7] S. M. Nicholls , R. Poplawski , M. J. Bull , et al., “Climb‐COVID: Continuous Integration Supporting Decentralised Sequencing for SARS‐CoV‐2 Genomic Surveillance,” Genome Biology 22, no. 1 (2021): 196. 34210356 10.1186/s13059-021-02395-y PMC8247108

[8] D. W. Wright , W. T. Harvey , J. Hughes , et al., “Tracking SARS‐CoV‐2 Mutations and Variants Through the COG‐UK‐Mutation Explorer,” Virus Evolution 8, no. 1 (2022): veac023. 35502202 10.1093/ve/veac023 PMC9037374

[9] D. Aggarwal , B. Warne , A. S. Jahun , et al., “Genomic Epidemiology of SARS‐CoV‐2 in a UK University Identifies Dynamics of Transmission,” Nature Communications 13, no. 1 (2022): 751. 10.1038/s41467-021-27942-w PMC8826310 35136068

[10] O. Eales , A. J. Page , L. de Oliveira Martins , et al., “SARS‐CoV‐2 Lineage Dynamics in England From September to November 2021: High Diversity of Delta Sub‐Lineages and Increased Transmissibility of AY. 4.2,” BMC Infectious Diseases 22, no. 1 (2022): 647. 35896970 10.1186/s12879-022-07628-4 PMC9326417

[11] J. T. McCrone , V. Hill , S. Bajaj , et al., “Context‐Specific Emergence and Growth of the SARS‐CoV‐2 Delta Variant,” Nature 610, no. 7930 (2022): 154–160. 35952712 10.1038/s41586-022-05200-3 PMC9534748

[12] H. Allen , A. Vusirikala , J. Flannagan , et al., “Household Transmission of COVID‐19 Cases Associated With SARS‐CoV‐2 Delta Variant (B. 1.617. 2): National Case‐Control Study,” Lancet Regional Health‐Europe 12 (2022): 100252. 34729548 10.1016/j.lanepe.2021.100252 PMC8552812

[13] O. Stirrup , F. Boshier , C. Venturini , et al., “SARS‐CoV‐2 Lineage B. 1.1. 7 Is Associated With Greater Disease Severity Among Hospitalised Women but not Men: Multicentre Cohort Study,” BMJ Open Respiratory Research 8, no. 1 (2021): e001029. 10.1136/bmjresp-2021-001029 PMC8453594 34544733

[14] S. A. Wilkinson , A. Richter , A. Casey , et al., “Recurrent SARS‐CoV‐2 Mutations in Immunodeficient Patients,” Virus Evolution 8, no. 2 (2022): veac050. 35996593 10.1093/ve/veac050 PMC9384748

[15] T. Crocker‐Buque , S. Williams , A. R. Brentnall , et al., “The Barts Health NHS Trust COVID‐19 Cohort: Characteristics, Outcomes and Risk Scoring of Patients in East London,” International Journal of Tuberculosis and Lung Disease 25, no. 5 (2021): 358–366. 10.5588/ijtld.20.0926 33977903

[16] A. Rambaut , E. C. Holmes , Á. O'Toole , et al., “A Dynamic Nomenclature Proposal for SARS‐CoV‐2 Lineages to Assist Genomic Epidemiology,” Nature Microbiology 5, no. 11 (2020): 1403–1407. 10.1038/s41564-020-0770-5 PMC7610519 32669681

[17] Barts Health NHS Trust COVID 19 EHR Extracted Dataset , (2023), https://web.www.healthdatagateway.org/dataset/d6e8163f-2ab8-4880-bbb6-a174b4a95632.

[18] S. R. Knight , A. Ho , R. Pius , et al., “Risk Stratification of Patients Admitted to Hospital With Covid‐19 Using the ISARIC WHO Clinical Characterisation Protocol: Development and Validation of the 4C Mortality Score,” BMJ (Clinical Research ed.) 370 (2020): 3339. 10.1136/bmj.m3339 PMC7116472 32907855

[19] R. K. Gupta , E. M. Harrison , A. Ho , et al., “Development and Validation of the ISARIC 4C Deterioration Model for Adults Hospitalised With COVID‐19: A Prospective Cohort Study,” Lancet Respiratory Medicine 9, no. 4 (2021): 349–359. 33444539 10.1016/S2213-2600(20)30559-2 PMC7832571

[20] M. Myrstad , H. Ihle‐Hansen , A. A. Tveita , et al., “National Early Warning Score 2 (NEWS2) on Admission Predicts Severe Disease and In‐Hospital Mortality From Covid‐19–A Prospective Cohort Study,” Scandinavian Journal of Trauma, Resuscitation and Emergency Medicine 28 (2020): 66. 32660623 10.1186/s13049-020-00764-3 PMC7356106

[21] Royal College of Physicians , National Early Warning Score (NEWS) 2: Standardising the Assessment of Acute‐Illness Severity in the NHS. Updated Report of a Working Party (RCP, 2017).

[22] Royal College of Physicians , National Early Warning Score (NEWS): Standardising the Assessment of Acute‐Illness Severity in the NHS. Report of a Working Party (RCP, 2012).

[23] C. N. Klabunde , A. L. Potosky , J. M. Legler , and J. L. Warren , “Development of a Comorbidity Index Using Physician Claims Data,” Journal of Clinical Epidemiology 53, no. 12 (2000): 1258–1267. 11146273 10.1016/s0895-4356(00)00256-0

[24] D. J. Grint , K. Wing , E. Williamson , et al., “Case Fatality Risk of the SARS‐CoV‐2 Variant of Concern B. 1.1. 7 in England, 16 November to 5 February,” Eurosurveillance 26, no. 11 (2021): 2100256. 33739254 10.2807/1560-7917.ES.2021.26.11.2100256 PMC7976383

[25] T. Nyberg , K. A. Twohig , R. J. Harris , et al., “Risk of Hospital Admission for Patients With SARS‐CoV‐2 Variant B. 1.1. 7: Cohort Analysis,” BMJ 373 (2021): n1412. 34130987 10.1136/bmj.n1412 PMC8204098

[26] P. Bager , J. Wohlfahrt , J. Fonager , et al., “Risk of Hospitalisation Associated With Infection With SARS‐CoV‐2 Lineage B. 1.1. 7 in Denmark: An Observational Cohort Study,” Lancet Infectious Diseases 21, no. 11 (2021): 1507–1517. 34171231 10.1016/S1473-3099(21)00290-5 PMC8219488

[27] N. G. Davies , C. I. Jarvis , W. J. Edmunds , N. P. Jewell , K. Diaz‐Ordaz , and R. H. Keogh , “Increased Mortality in Community‐Tested Cases of SARS‐CoV‐2 Lineage B. 1.1. 7,” Nature 593, no. 7858 (2021): 270–274. 33723411 10.1038/s41586-021-03426-1 PMC9170116

[28] E. Volz , S. Mishra , M. Chand , et al., “Assessing Transmissibility of SARS‐CoV‐2 Lineage B. 1.1. 7 in England,” Nature 593, no. 7858 (2021): 266–269. 33767447 10.1038/s41586-021-03470-x

[29] N. G. Davies , S. Abbott , R. C. Barnard , et al., “Estimated Transmissibility and Impact of SARS‐CoV‐2 Lineage B. 1.1. 7 in England,” Science 372, no. 6538 (2021): eabg3055. 33658326 10.1126/science.abg3055 PMC8128288

[30] Department of Health and Social Care . UK COVID‐19 Vaccines Delivery Plan. London: UK Government, https://assets.publishing.service.gov.uk/government/uploads/system/uploads/attachment_data/file/951928/uk-covid-19-vaccines-delivery-plan-final.pdf, 2025.

[31] J. L. Bernal , N. Andrews , C. Gower , et al., “Effectiveness of the Pfizer‐Biontech and Oxford‐Astrazeneca Vaccines on Covid‐19 Related Symptoms, Hospital Admissions, and Mortality in Older Adults in England: Test Negative Case‐Control Study,” BMJ 373 (2021): n1088. 33985964 10.1136/bmj.n1088 PMC8116636

[32] S. Pei , S. Kandula , and J. Shaman , “Differential Effects of Intervention Timing on COVID‐19 Spread in the United States,” Science Advances 6, no. 49 (2020): eabd6370. 33158911 10.1126/sciadv.abd6370 PMC7821895

[33] K. E. Mason , G. Maudsley , P. McHale , A. Pennington , J. Day , and B. Barr , “Age‐Adjusted Associations Between Comorbidity and Outcomes of COVID‐19: A Review of the Evidence From the Early Stages of the Pandemic,” Frontiers in Public Health 9 (2021): 584182. 34422736 10.3389/fpubh.2021.584182 PMC8377370

