# Supplementary material for: Analysis of Variants' Dynamic Using the CLIMB Database in COVID‐19 Patients Admitted to Hospitals of Barts Health NHS Trust
Source: J Med Virol. 2025 May 24;97(5):e70402. doi: 10.1002/jmv.70402 (PMC12102684; doi:10.1002/jmv.70402)
Supplement: Supplementary file 1 — Supplementary material. [file JMV-97-e70402-s001.docx]

**Analysis of variants’ dynamic using the CLIMB database in COVID-19 patients admitted to hospitals of Barts Health NHS Trust**

Concetta Piazzese^1,2,3^, Sophie Williams^1,2,3^, Adam Brentall^4^, Beatrix Kele^5^, Jon Bible^6^, Kathryn Harris^6^, Teresa Cutino-Moguel^6^

^1^ Barts Life Sciences, Barts Health NHS Trust, London, United Kingdom.

^2^ PHURI, Queen Mary University of London, London, United Kingdom.

^3^ DERI, Queen Mary University of London, London, United Kingdom.

^4^ Wolfson Institute of Population Health, Queen Mary University of London, London, United Kingdom.

^5^ Respiratory Virus Unit, UK Health Security Agency, London, United Kingdom.

^6^ Department of Virology, Division of Infection, Barts Health NHS Trust, London, United Kingdom.

**Supplementary material**


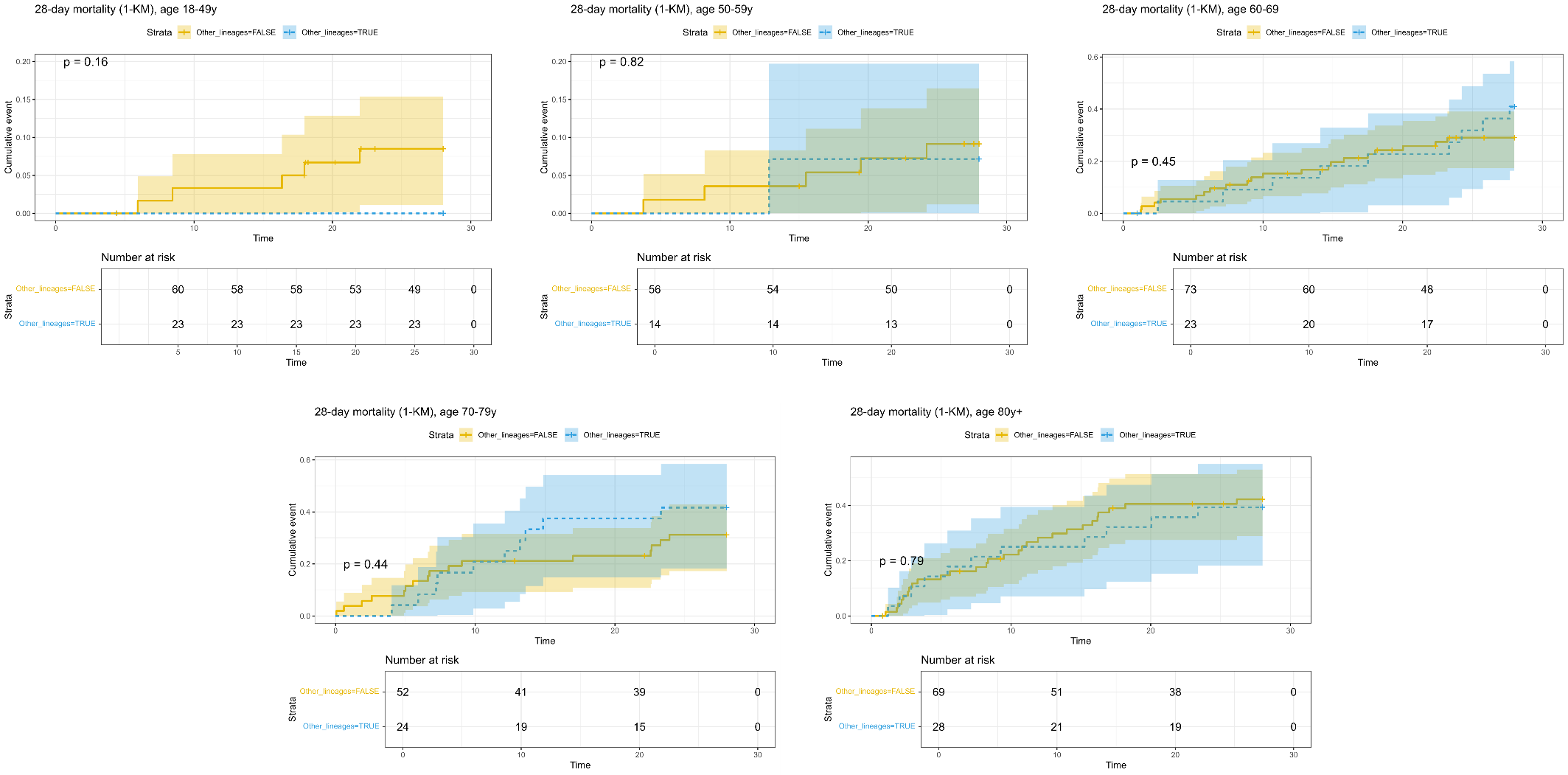


Figure F1. Kaplan-Meier survival curves and risk tables illustrating the 28-day mortality rates for COVID-19 patients stratified by age group and viral lineages. The survival probabilities are plotted for lineage Alpha (B.1.1.7) and other lineages across different age categories: 18-49 years, 50-59 years, 60-69 years, 70-79 years, and 80+ years.


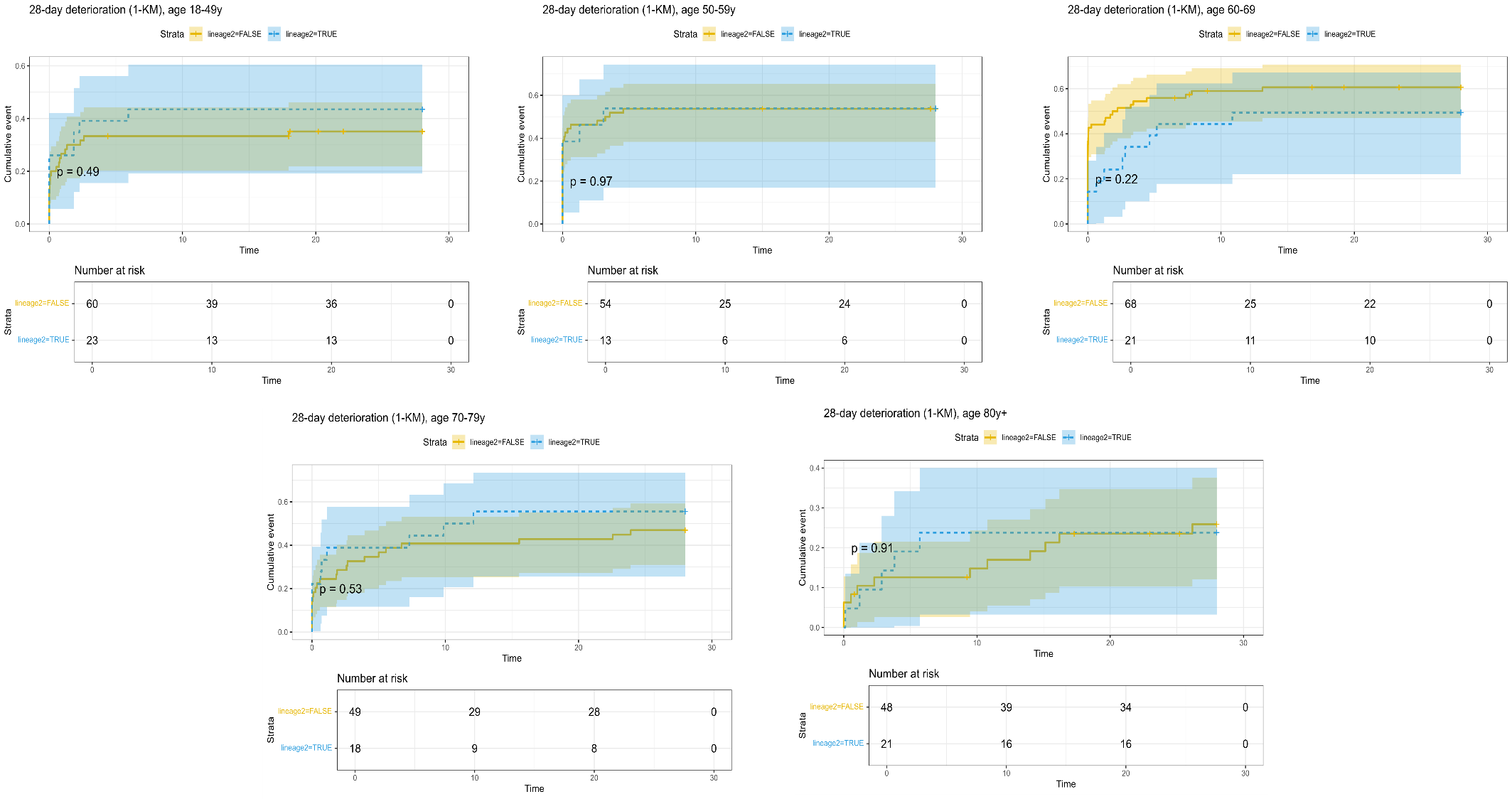


Figure F2. Kaplan-Meier curves and risk tables depicting the 28-day deterioration rates, defined as ICU admission or death, for COVID-19 patients by age group and viral lineage. The analysis includes lineage Alpha (B.1.1.7) and other lineages for the age categories of 18-49 years, 50-59 years, 60-69 years, 70-79 years, and 80+ years.

Table S1. Adjusted estimates and confidence intervals for deterioration outcomes among different ethnic groups, with adjustments for the ISARIC 4C-D prognostic score. The table underscores the variability in deterioration risks, particularly notable in the Unknown entry group with lineage Alpha.

| Parameter | Estimate (95%CI) |
| --- | --- |
| 4CD, Ethnicity A – White, entry (Lineage Alpha) | 0.75 (0.50 to 1.01) |
| 4CD, Ethnicity A – White, subsq (Lineage Alpha) | 0.48 (0.16 to 0.80) |
| 4CD, Ethnicity A – White, entry (Other lineages) | 0.79 (0.35 to 1.24) |
| 4CD, Ethnicity A – White, subsq (Other lineages) | 0.53 (-0.04 to 1.11) |
| 4CD, Ethnicity B – Asian, entry (Lineage Alpha) | 2.04 (0.91 to 5.29) |
| 4CD, Ethnicity B – Asian, subsq (Lineage Alpha) | 0.86 (0.29 to 2.84) |
| 4CD, Ethnicity B – Asian, entry (Other lineages) | 2.03 (0.75 to 6.29) |
| 4CD, Ethnicity B – Asian, subsq (Other lineages) | 1.60 (0.60 to 4.82) |
| 4CD, Ethnicity C – Black, Mixed or Other, entry (Lineage Alpha) | 2.06 (0.72 to 6.95) |
| 4CD, Ethnicity C – Black, Mixed or Other subsq (Lineage Alpha) | 1.14 (0.49 to 4.05) |
| 4CD, Ethnicity C – Black, Other, & Mixed, entry (Other lineages) | 0.94 (0.12 to 4.18) |
| 4CD, Ethnicity C – Black, Other, & Mixed, subsq (Other lineages) | 1.98 (0.42 to 9.38) |
| Ethnicity G - Unknown, entry (Lineage Alpha) | 2.80 (1.29 to 6.05) |
| Ethnicity G – Unknown, subsq (Lineage Alpha) | 0.57 (0.13 to 2.47) |
| Ethnicity G – Unknown, entry (Other lineages) | 0.00 (0.00 to Inf) |
| Ethnicity G – Unknown, subsq (Other lineages) | 2.01 (0.25 to 16.07) |

Table S2. Summary statistics for continuous variables across lineages, showing means, medians, standard deviations (SD), minimum (Min), lower quartile (LQ), upper quartile (UQ), maximum (Max), percentage of unknown values, unadjusted p-values (pval), and age-adjusted p-values (pval2), calculated using the Wilcoxon test for non-parametric comparison for two independent groups (Lineage Alpha vs other lineages).

|  | Mean | Median | SD | Min | LQ | UQ | Max | Unknown | pval | pval2 |
| --- | --- | --- | --- | --- | --- | --- | --- | --- | --- | --- |
| Age  (Lineage Alpha) | 64.3 | 66 | 17.2 | 18 | 52.5 | 77.5 | 96 | 0 (0.0%) | 0.47 |  |
| Age  (Other lineages) | 65.8 | 67 | 16.6 | 21 | 53 | 79.2 | 94 | 0 (0.0%) |  |  |
| Respiratory Rate (Lineage Alpha) | 23.7 | 22 | 6.3 | 13.1 | 19 | 26 | 48.5 | 11 (3.5%) | 0.005 | 0.22 |
| Respiratory Rate  (Other lineages) | 22.7 | 20 | 7.9 | 16 | 18 | 23.6 | 67 | 14 (12.5%) |  |  |
| Urea  (Lineage Alpha)) | 9.4 | 6 | 8 | 1 | 4 | 11 | 42 | 30 (9.6%) | 0.49 | <0.001 |
| Urea  (Other lineages) | 9.4 | 7 | 7 | 3 | 5 | 12 | 33 | 27 (24.1%) |  |  |
| C-Reactive Protein  (Lineage Alpha) | 101.2 | 75 | 81.7 | 0 | 39 | 146 | 373 | 54 (17.4%) | 0.6 | 0.6 |
| C-Reactive Protein  (Other lineages) | 97.4 | 67 | 86.6 | 1 | 39.5 | 126.5 | 363 | 37 (33.0%) |  |  |
| Oxygen Saturation on Air  (Lineage Alpha) | 93.6 | 95 | 6 | 52 | 92.6 | 96.5 | 100 | 132 (42.4%) | 0.009 | 0.1 |
| Oxygen Saturation on Air (Other lineages) | 95.1 | 96 | 4 | 81 | 94 | 97.5 | 100 | 49 (43.8%) |  |  |
| Oxygen Saturation (Lineage Alpha) | 94.7 | 95 | 2.7 | 74.3 | 93.5 | 96.2 | 100 | 12 (3.9%) | 0.044 | 0.044 |
| Oxygen Saturation (Other lineages) | 95.1 | 96 | 3.3 | 75 | 93.8 | 97 | 100 | 13 (11.6%) |  |  |
| Creatinine  (Lineage Alpha) | 192.8 | 98 | 259.9 | 11 | 72 | 148 | 1819 | 34 (10.9%) | 0.44 | <0.001 |
| Creatinine  (Other lineages) | 165.9 | 104 | 210.3 | 26 | 77.5 | 162.5 | 1527 | 30 (26.8%) |  |  |
| Albumim  (Lineage Alpha) | 37 | 37 | 4.6 | 20 | 34 | 40 | 51 | 42 (13.5%) | 0.034 | 0.041 |
| Albumim  (Other lineages) | 38 | 39 | 4.7 | 21 | 36 | 41 | 49 | 33 (29.5%) |  |  |
| Systolic Blood Pressure (Lineage Alpha) | 130.2 | 127 | 21.8 | 78 | 114.6 | 144 | 212.5 | 11 (3.5%) | 0.27 | <0.001 |
| Systolic Blood Pressure (Other lineages) | 133.2 | 129.4 | 21 | 100 | 119.5 | 141 | 231.2 | 14 (12.5%) |  |  |
| Diastolic Blood Pressure (Lineage Alpha) | 73.9 | 73 | 11.2 | 39 | 66.9 | 81 | 111 | 11 (3.5%) | 0.27 | 0.9 |
| Diastolic Blood Pressure (Other lineages) | 75.5 | 75 | 10.8 | 53 | 67.8 | 81.8 | 107 | 14 (12.5%) |  |  |
| NCI Comorbidity Index (Lineage Alpha) | 1.8 | 1 | 2 | 0 | 0 | 3 | 8 | 0 (0.0%) | 0.021 | <0.001 |
| NCI Comorbidity Index (Other lineages) | 2.3 | 2 | 2.2 | 0 | 0 | 4 | 9 | 0 (0.0%) |  |  |
| Neutrophil  (Lineage Alpha) | 6.2 | 6 | 3.4 | 0 | 4 | 8 | 19 | 28 (9.0%) | 0.13 | 0.8 |
| Neutrophil  (Other lineages) | 5.5 | 5 | 2.9 | 0 | 4 | 7 | 15 | 26 (23.2%) |  |  |
| Lymphocyte Count (Lineage Alpha) | 1 | 0.9 | 0.6 | 0 | 0.7 | 1.2 | 5.1 | 28 (9.0%) | 0.7 | 0.13 |
| Lymphocyte Count (Other lineages) | 1.1 | 0.9 | 1 | 0.3 | 0.7 | 1.3 | 9 | 26 (23.2%) |  |  |
| NEWS (Lineage Alpha) | 4.8 | 5 | 2.6 | 0 | 3 | 6.5 | 14 | 18 (5.8%) | 0.004 | 0.16 |
| NEWS (Other lineages) | 3.9 | 3.5 | 2.9 | 0 | 1 | 6 | 11 | 1 (0.9%) |  |  |
| Pulse Rate  (Lineage Alpha) | 87.2 | 87 | 16.7 | 47 | 75 | 98.1 | 129 | 43 (13.8%) | 0.6 | <0.001 |
| Pulse Rate  (Other lineages)) | 86.4 | 85.9 | 16.2 | 49 | 75 | 97.8 | 132 | 20 (17.9%) |  |  |
| Temperature (Lineage Alpha) | 37.3 | 36.9 | 6.4 | 33.4 | 36.5 | 37.4 | 147 | 15 (4.8%) | 0.8 | 0.19 |
| Temperature (Other lineages) | 37 | 36.8 | 0.7 | 35.4 | 36.4 | 37.4 | 39.1 | 17 (15.2%) |  |  |

Table S3. Summary statistics proportions for various binary variables across two lineage groups (Lineage Alpha vs other lineages), including proportions (%), counts and percentages of unknown values, unadjusted p-values (pval) and age-adjusted p-values (pval2), calculated using the Chi-square tests for categorical data. The table consistently reports a 0% proportion of individuals with "Unknown" status across both lineage groups for all variables, indicating complete data availability and reliability in the analysis. GCS-AVPU, Glasgow Coma Scale – Alert, Verbal, Pain, Unresponsive.

|  | Proportion | Unknown | pval | pval2 |
| --- | --- | --- | --- | --- |
| GCS-AVPU (Lineage Alpha) | 49/300 (16%) | 11/311 (4%) | 0.6 | 0.23 |
| GCS-AVPU (Other lineages) | 13/98 (13%) | 14/112 (12%) |  |  |
| Cardiovascular Disease (Lineage Alpha) | 63/311 (20%) | 0/311 (0%) | 1 | 0.9 |
| Cardiovascular Disease (Other lineages) | 23/112 (21%) | 0/112 (0%) |  |  |
| Congestive Heart Failure (Lineage Alpha) | 51/311 (16%) | 0/311 (0%) | 0.5 | 0.6 |
| Congestive Heart Failure (Other lineages) | 22/112 (20%) | 0/112 (0%) |  |  |
| Peripheral Artery Disease (Lineage Alpha) | 5/311 (2%) | 0/311 (0%) | 0.9 | 0.5 |
| Peripheral Artery Disease (Other lineages) | 1/112 (1%) | 0/112 (0%) |  |  |
| Dementia (Lineage Alpha) | 21/311 (7%) | 0/311 (0%) | 0.8 | 0.9 |
| Dementia (Other lineages) | 9/112 (8%) | 0/112 (0%) |  |  |
| Respiratory Disease (Lineage Alpha) | 28/311 (9%) | 0/311 (0%) | 0.16 | 0.18 |
| Respiratory Disease (Other lineages) | 16/112 (14%) | 0/112 (0%) |  |  |
| Mild Liver Disease (Lineage Alpha) | 25/311 (8%) | 0/311 (0%) | 0.23 | 0.19 |
| Mild Liver Disease (Other lineages) | 14/112 (12%) | 0/112 (0%) |  |  |
| Severe Liver Disease (Lineage Alpha) | 13/311 (4%) | 0/311 (0%) | 0.097 | 0.071 |
| Severe Liver Disease (Other lineages) | 10/112 (9%) | 0/112 (0%) |  |  |
| Diabetes Mellitus with Complications (Lineage Alpha) | 91/311 (29%) | 0/311 (0%) | 0.03 | 0.037 |
| Diabetes Mellitus with Complications (Other lineages) | 46/112 (41%) | 0/112 (0%) |  |  |
| Diabetes Mellitus Complications (Lineage Alpha) | 85/311 (27%) | 0/311 (0%) | 0.17 | 0.19 |
| Diabetes Mellitus Complications (Other lineages) | 39/112 (35%) | 0/112 (0%) |  |  |
| Chronic Kidney Disease (Lineage Alpha) | 85/311 (27%) | 0/311 (0%) | 0.31 | 0.34 |
| Chronic Kidney Disease (Other lineages) | 37/112 (33%) | 0/112 (0%) |  |  |
| Malignancy with High-Dose Therapy (Lineage Alpha) | 13/311 (4%) | 0/311 (0%) | 0.5 | 0.45 |
| Malignancy with High-Dose Therapy (Other lineages) | 7/112 (6%) | 0/112 (0%) |  |  |
| Metabolic Disease (Lineage Alpha) | 8/311 (3%) | 0/311 (0%) | 0.19 | 0.036 |
| Metabolic Disease (Other lineages) | 0/112 (0%) | 0/112 (0%) |  |  |
| Lymphoma (Lineage Alpha) | 3/311 (1%) | 0/311 (0%) | 0.9 | 0.6 |
| Lymphoma (Other lineages) | 2/112 (2%) | 0/112 (0%) |  |  |
| Immunosuppression (Lineage Alpha) | 2/311 (1%) | 0/311 (0%) | 0.6 | 0.29 |
| Immunosuppression (Other lineages) | 2/112 (2%) | 0/112 (0%) |  |  |
| Connective Tissue Disease (Lineage Alpha) | 9/311 (3%) | 0/311 (0%) | 0.6 | 0.5 |
| Connective Tissue Disease (Other lineages) | 5/112 (4%) | 0/112 (0%) |  |  |
| Leukaemia (Lineage Alpha) | 6/311 (2%) | 0/311 (0%) | 0.9 | 0.7 |
| Leukaemia (Other lineages) | 3/112 (3%) | 0/112 (0%) |  |  |
| Stroke (Lineage Alpha) | 25/311 (8%) | 0/311 (0%) | 0.14 | 0.14 |
| Stroke (Other lineages) | 15/112 (13%) | 0/112 (0%) |  |  |
| Hemiplegia (Lineage Alpha) | 9/311 (3%) | 0/311 (0%) | 0.6 | 0.5 |
| Hemiplegia (Other lineages) | 5/112 (4%) | 0/112 (0%) |  |  |
| Peptic Ulcer Disease (Lineage Alpha) | 5/311 (2%) | 0/311 (0%) | 0.9 | 0.7 |
| Peptic Ulcer Disease (Other lineages) | 1/112 (1%) | 0/112 (0%) |  |  |
| Diabetes Mellitus (Lineage Alpha) | 113/311 (36%) | 0/311 (0%) | 0.078 | 0.098 |
| Diabetes Mellitus (Other lineages) | 52/112 (46%) | 0/112 (0%) |  |  |
| Hypertension (Lineage Alpha) | 173/311 (56%) | 0/311 (0%) | 0.25 | 0.29 |
| Hypertension (Other lineages) | 70/112 (62%) | 0/112 (0%) |  |  |
| Systemic Rheumatic Disease (Lineage Alpha) | 31/311 (10%) | 0/311 (0%) | 0.28 | 0.29 |
| Systemic Rheumatic Disease (Other lineages) | 16/112 (14%) | 0/112 (0%) |  |  |
| On Air (Lineage Alpha) | 120/299 (40%) | 12/311 (4%) | 0.6 | 0.6 |
| On Air (Other lineages) | 36/99 (36%) | 13/112 (12%) |  |  |
